# Enhancing capability for continuous organisational improvement and learning in healthcare organisations: a systematic review of the literature 2013–2022

**DOI:** 10.1136/bmjoq-2023-002566

**Published:** 2024-04-02

**Authors:** Ninni Löfqvist

**Affiliations:** 1 Department of Communication, Quality Management, and Information Systems, Mid Sweden University, Östersund, Sweden; 2 Department of Pediatrics, Region Västernorrland, Härnösand, Sweden

**Keywords:** Continuous quality improvement, Healthcare quality improvement, Quality improvement

## Abstract

**Background:**

Healthcare organisations strive to meet their current and future challenges and need to increase their capacity for continuous organisational improvement and learning (COIL). A key aspect of this capacity is the development of COIL capability among employees.

**Objective:**

This systematic review aims to explore common attributes of interventions that contribute to the development of COIL capability in healthcare organisations and to explore possible facilitating and hindering factors.

**Methods:**

A comprehensive search was conducted in Scopus, MEDLINE and Business Source Complete for primary research studies in English or Swedish, in peer-reviewed journals, focusing on organisational improvements and learning in healthcare organisations. Studies were included if they were published between 2013 and 23 November 2022, reported outcomes on COIL capability, included organisations or groups, and were conducted in high-income countries. The included articles were analysed to identify themes related to successful interventions and factors influencing COIL capability.

**Results:**

Thirty-six articles were included, with two studies reporting unsuccessful attempts at increasing COIL capability. The studies were conducted in nine different countries, encompassing diverse units, with the timeframes varying from 15 weeks to 8 years, and they employed quantitative (n=10), qualitative (n=11) and mixed methods (n=15). Analysis of the included articles identified four themes for both attributes of interventions and the factors that facilitated or hindered successful interventions: (1) engaged managers with a strategic approach, (2) external training and guidance to develop internal knowledge, skills and confidence, (3) process and structure to achieve improvements and learning and (4) individuals and teams with autonomy, accountability, and safety.

**Conclusion:**

This review provides insights into the intervention attributes that are associated with increasing COIL capability in healthcare organisations as well as factors that can have hindering or facilitating effects. Strategic management, external support, structured processes and empowered teams emerged as key elements for enhancing COIL capability.

**WHAT IS ALREADY KNOWN ON THIS TOPIC**

- Continuous improvement is commonly used in healthcare, and the need for organisational learning is recognised. It is essential for healthcare organisations to build the capability among staff to achieve continuous organisational improvement and learning (COIL), but there is limited research on how to develop this capability.

**WHAT THIS STUDY ADDS**

- This study provides insights about attributes that have been associated with building COIL capability in healthcare organisations in previous research and adds to the knowledge about potential facilitating and hindering factors.

**HOW THIS STUDY MIGHT AFFECT RESEARCH, PRACTICE AND/OR POLICY**

- This review’s findings suggest that strategic management, external support, structured processes and empowered teams are key aspects of enhancing COIL capability, and that further research is needed as to their interactions and the importance of the various attributes and possible affecting factors in practice.

## Introduction

The healthcare sector faces great challenges when aiming for quality of care and health services that are effective, safe, people centred, timely, equitable, integrated, and efficient.^1^

Some of these challenges are related to the need for healthcare to adapt to new situations such as emerging epidemics/pandemics, antibiotic resistance, climate change^2^ and taking care of an increasingly ageing population with an increase in the burden from chronic diseases.^3^ Other challenges concern handling technical and medical innovations without increasing costs,^4^ implementing evidence-based medicine and practices^5^ and providing a positive working environment to prevent health issues among healthcare employees.^6^ Another big challenge is decreasing the occurrence of patient harm caused by preventable adverse events, since they cause unnecessary patient harm, and since approximately 15% of hospitals’ total activity consists of adverse events with a large amount of these being considered preventable.^7^

All these challenges require improvements of the systems and processes of healthcare,^8^ with a focus on the entire system and its different aspects—including, for example, the emotional experience and well-being of the employees.^9^ This need for improvement is recognised by Nundy *et al* 10 as a quintuple aim for healthcare: improving population health, health equity, enhancing the care experience, reducing costs and improving the work environment among healthcare workers. It is also recognised in legislation—for example, in Sweden, where the Swedish Health and Medical Care Act (SFS 2017:30) demands that ‘the quality of operations shall systematically and continuously be developed and secured’.^11^

Systematic and continuous development can be achieved through continuous improvement (CI), which is commonly used in healthcare.^12^ CI is derived from the area of quality management (QM), where the intent is to gradually increase the organisational knowledge through repeated cycles of small tests of learning by alternating between thought and action.^13^

Learning is needed in order for CI to generate real and sustainable improvement.^14 15^ Furthermore, the improvements need to focus not only on products, services and processes but also on the way people learn and improve together, that is, organisational learning (OL).^16^ OL has been defined as ‘a process of positive change in an organization’s collective knowledge, cognition and actions, which enhances the organization’s ability to achieve its desired outcomes’ 17 (p643). Despite this close connection between CI and OL, a need for future research that focuses on how QM can enhance OL has been identified.^18^

The success of CI has so far been limited, proposedly due to a lack of focus on aspects of OL.^13 14 19^ Much focus is spent on implementing specific methods and techniques, and even high-performing companies focus a lot of their CI on firefighting activities.^20^ Reflection and learning are not always embedded in the CI work, making it hard to achieve OL.^19^

To emphasise the need for OL together with CI, the term continuous organisational improvement and learning (COIL), as stated by Nyström *et al*,^21^ will be used in this article.

Building enough capacity for improvement and learning requires organisations to have the associated capabilities among a sufficient number of their employees, at the right levels of the organisation,^22^ but there is limited research on how to develop this capability.^23^ Having capability means being able to perform the actions that are needed to reach desired goals.^24^ Capability for COIL is here defined as having the knowledge, skills, abilities and confidence to make improvements within the organisation and promote OL. This includes, but is not limited to, the ability to communicate about concerns, solve problems, learn from errors, collaborate, and use quality improvement principles.^25^

In a recent review, Loper *et al* 26 studied capacity building for continuous quality improvements in healthcare, medical education and public health from an implementation science perspective. The authors used the definition ‘a culture of sustained improvement targeting the elimination of waste in all systems and processes of an organisation’ and focused their search on articles including ‘quality’ but not ‘learning’. They present a framework of guiding principles and core components for supporting continuous quality improvement capacity building and suggest that further research is needed regarding how to build organisational capacity and how these efforts are affected by contextual factors.^26^ This is in line with Fundin *et al* 18 and Kaplan *et al* 27 calls for research on how QI initiatives are influenced by different contexts.

This study tries to answer the call for research on how healthcare organisations can build capability for COIL, as an essential part of overall capacity, and considers the possible influence of contextual factors. It also contributes to the knowledge about connections between QM and OL by looking at the concept of COIL where both the aspects of CI and learning in organisations are highlighted.

## Aim

The aim of this study is to explore common attributes of interventions that contribute to the development of COIL capability in healthcare organisations and to explore possible facilitating or hindering factors.

## Method

This systematic review followed recognised guidance and reporting.^28^

### Patient and public involvement

It was not appropriate or possible to involve patients or the public in the design, conduct, reporting, or dissemination plans of this study.

### Search strategy

The search was conducted in three electronic databases: Scopus, MEDLINE (via EBSCO) and Business Source Complete (via EBSCO). Searches in all three databases used the same combination of keywords, which were related to healthcare organisations, CIs and OL. See box 1 for a complete list of the keywords. All databases were searched from their inception to 23 November 2022 and were limited to studies published in English or Swedish in peer-reviewed journals.

Box 1 **Keywords used in database searches**

"health care" OR healthcare OR "health service*" OR "medical service*" OR hospital* OR "medical care" OR clinic OR "primary care" OR "urgent care" OR nurs*

AND

"quality improvement*" OR "continuous improvement*" OR "improvement capabilit*" OR "capability of improvement*" OR "improvement approach*" OR "improvement science" OR "improvement knowledge" OR "improvement strateg*" OR "total quality management" OR "quality management" OR "change management" OR "safety management" OR "quality culture" OR "safety culture" OR kaizen OR pdsa OR pdca OR "plan-do-study" OR "plan-do-check" OR kata

AND

"learning organization*" OR "learning healthcare" OR "learning health care" OR "learning health system*" OR "group learning" OR "team learning" OR "organizational learning" OR "collaborative learning" OR "system* learning" OR "organizational development" OR "organizational improvement" OR "organizational change" OR "organizational behavior management" OR "double-loop learning" OR "action learning" OR "after-action review" OR "forward-looking learning" OR "practical learning" OR "systematic learning" OR coaching OR grow

### Eligibility criteria

Studies included those published in peer-reviewed journals (confirmed using Ulrichsweb) written in the English or Swedish languages and met the following inclusion criteria: published between 2013 and 2022; conducted in countries in Europe, North America, Australia or New Zealand; and studied an intervention aiming at achieving organisational improvements and/or learning in healthcare organisations.

Studies were excluded if they were non-primary studies; conducted in a non-healthcare setting; focused only on improvement or learning for individuals and not groups of people or organisations and did not report results related to COIL capability.

This study focused on research that has been conducted recently—therefore, articles published before 2013 were excluded. Also, the study was limited to high-income countries that face similar challenges in healthcare.^29^

### Quality assessment

Depending on the purpose of the review, the researcher needs to decide on whether to use quality appraisal to exclude reports from the review or to keep them, and then assess and describe the quality of the reports as part of the results.^30^ In this study, all reports were published in peer-reviewed journals and consisted of empirical studies. Since the purpose of the study was to assess attributes and enabling or hindering factors for the interventions, reports were assessed regarding the clarity and transparency of their descriptions of the interventions. All included reports were assessed as having enough clarity and transparency to be included in the review.

### Screening

**Figure 1:**
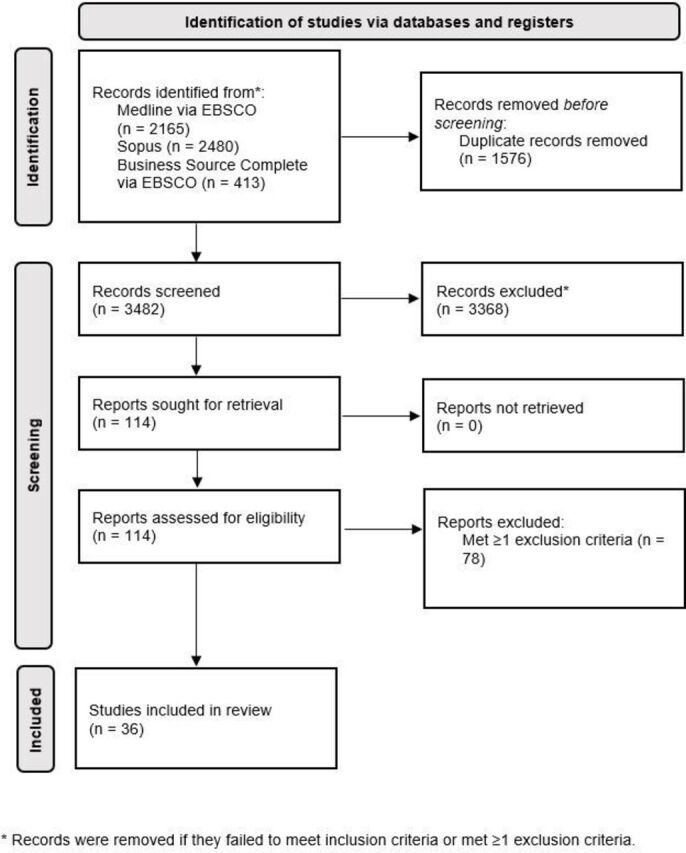
Literature flowchart.

All articles were exported to EndNote. See figure 1 for a flowchart of the screening process. Duplicates were removed. The author screened the titles and abstracts to remove articles that did not meet the inclusion criteria, or which met any of the exclusion criteria. The remaining articles were retrieved in full text and screened further with respect to the eligibility criteria for inclusion or exclusion.

### Data extraction and synthesis

Thirty-six articles were included in the study and are presented in online supplemental appendix 1. In table 1, the interventions from the studies are presented. All studies except the ones by Petit dit Dariel *et al* 31 and Waring and Crompton^32^ reported on improvements in COIL capability. Therefore, 34 articles were included in the data extraction and synthesis concerning attributes of interventions that were successful in increasing COIL capability, while attributes from the remaining two^31 32^ were analysed separately. All studies were included in the data extraction and synthesis concerning factors that hinder or facilitate the interventions.

10.1136/bmjoq-2023-002566.supp1 Supplementary data

**Table 1:** Overview of the interventions used in the studies

Author (year)	Intervention
Glasheen *et al* (2022)^62^	Institute for Health care Quality, Safety and Efficiency (IHQSE)
Källman *et al* (2022)^41^	Green Cross instead of other incident reporting system
Myren *et al* (2022)^56^	Using a cyclic workflow (PDCA) to improve implementation
Peet *et al* (2022)^53^	Emancipatory Practice Development (ePD)
Sweeney *et al* (2022)^48^	More effective facilitators compared to less effective
Choo *et al* (2021)^57^	Reflection on surprises during QI projects (Design for Six Sigma)
Damschroder *et al* (2021)^63^	LEAP programme to increase frontline teams' quality improvement capability
Penney *et al* (2021)^49^	Distance coaching vs no coaching
Steensgaard *et al* (2021)^40^	An action research process to engage nurses in development
Walunas *et al* (2021)^50^	Different activities and strategies for practice facilitation
Arora *et al* (2020)^44^	Improving Clinical Flow Collaborative
Lefebvre *et al* (2020)^46^	Education programme (including medico-legal risk)
Petit dit Dariel and Cristofalo (2020)^31^	PACTE (Programme d’amélioration continue du travail en équipe), an experimental programme aimed at improving teamwork
Rattray *et al* (2020)^47^	A virtual ‘Hub’ dashboard that provide performance data, a resource library, and a forum for sharing QI plans and tools
Sarff and O'Brien (2020)^37^	Quality Academy Program
Dixon and Wellsteed (2019)^64^	To emphasise the need for teamwork on a QI project, stages in the QI model were explained using an acronym, A–TEAM.
Gerrish *et al* (2018)^42^	The clinical microsystems (CMS) methodology to develop an integrated falls pathway
Nyström *et al* (2018)^21^	Sustainable Improvement and Development through Strategic and Systematic Approaches (SIDSSA)
Hefner *et al* (2017)^43^	Crew resource management (CRM)
Morgan *et al* (2017)^59^	Staff-led quality improvement intervention
Pannick *et al* (2017)^65^	Prospective clinical team surveillance (PCTS)
Rydenfält *et al* (2017)^34^	The krAft methodology
Stelson *et al* (2017)^36^	Employee-driven healthcare CI projects
Waring and Crompton (2017)^32^	Purposeful adoption of social movement ideas in the implementation of a QI strategy
McNamara *et al* (2016)^55^	On-site interdisciplinary QI learning collaborative
Restrepo *et al* (2016)^35^	The five-star process (STARS)
Tibor *et al* (2016)^33^	A collaborative learning approach for process improvement
Fieldston *et al* (2015)^38^	Innovation Units for embedding rapid-cycle improvement capabilities
Hellström *et al* (2015)^39^	Adopting a management innovation
McGrath and Blyke (2015)^51^	Performance improvement competency development programme: the Value Institute (VI)
Rangachari *et al* (2015)^52^	Periodic top-down QI communication
Simons *et al* (2015)^45^	Lean management
Day (2014)^61^	ENGAGE and focus group work
Nyström *et al* (2014)^60^	Two QI programmes being implemented at the same time
Fisher *et al* (2013)^58^	Engagement of Groups in Family Medicine Board Maintenance of Certification
Moule *et al* (2013)^54^	The Plan-Do-Study-Act (PDSA) model

The software OpenCode V.4.03 was used for data extraction, with information elements in the articles being coded inductively. A constant comparative method was used to synthesise the data and identify keywords across the studies. Primary data from individual studies were coded and compared by the author, generating initial concepts reflecting attributes of successful interventions and factors that hinder or facilitate interventions. Similar concepts were tentatively labelled with subcategories and categories for the attributes of interventions. Categories were then discussed by a research team consisting of the author, a PhD in QM, and a PhD in disability science, with further sifting, sorting and comparison within and between categories. The process finally resulted in four themes describing the attributes of interventions that were successful in increasing COIL capability. Because the codes and tentative categories for hindering and facilitating factors appeared to be very similar to the attributes the research team decided to deductively assign the codes for the factors to the same four themes that had been created for the attributes.

## Results

An overview of the included articles is found in online supplemental appendix 1. The studies were conducted in nine different countries, with most in the USA (n=16), UK (n=7) and Sweden (n=5). The units studied included single units or wards, several units, single hospitals, institutes or departments, medical centres/treatment teams, primary care practices also including individual physicians, multiple organisations and facilitators working with practices. Patients participated in two of the studies. The timeframe for the studies varied between 15 weeks and 8 years, and some were retrospective studies. There was a mix of quantitative (n=10), qualitative (n=11) and mixed methods studies (n=15). Methods for CIs and learning differed between the studies, with the plan-do-study/check-act (PDSA/PDCA) cycle being reported in 16 studies. Table 2 shows how many attributes, facilitating factors and hindering factors that were found in each study.

**Table 2:** Number of attributes, facilitating and hindering factors found in the articles

Author (year)	Attributes of interventions	Facilitating factors	Hindering factors
Glasheen *et al* (2022)^62^	13	10	0
Källman *et al* (2022)^41^	8	0	0
Myren *et al* (2022)^56^	11	11	1
Peet *et al* (2022)^53^	36	12	1
Sweeney *et al* (2022)^48^	16	4	1
Choo *et al* (2021)^57^	18	4	2
Damschroder *et al* (2021)^63^	24	1	2
Penney *et al* (2021)^49^	28	10	12
Steensgaard *et al* (2021)^40^	11	9	0
Walunas *et al* (2021)^50^	10	2	1
Arora *et al*. (2020)^44^	18	4	7
Lefebvre *et al*. (2020)^46^	13	7	7
Petit dit Dariel and Cristofalo (2020)^31^	16	0	21
Rattray *et al* (2020)^47^	18	5	1
Sarff and O'Brien (2020)^37^	15	9	0
Dixon and Wellsteed (2019)^64^	23	7	0
Gerrish *et al* (2018)^42^	30	16	7
Nyström *et al* (2018)^21^	47	31	11
Hefner *et al* (2017)^43^	10	5	7
Morgan *et al* (2017)^59^	15	7	0
Pannick *et al* (2017)^65^	22	9	8
Rydenfält *et al* (2017)^34^	24	0	1
Stelson *et al* (2017)^36^	10	5	10
Waring and Crompton (2017)^32^	15	3	8
McNamara *et al* (2016)^55^	18	9	0
Restrepo *et al* (2016)^35^	21	0	3
Tibor *et al* (2016)^33^	27	9	3
Fieldston *et al* (2015)^38^	26	16	3
Hellström *et al* (2015)^39^	38	10	0
McGrath and Blyke (2015)^51^	29	12	4
Rangachari *et al* (2015)^52^	16	2	0
Simons *et al* (2015)^45^	6	7	0
Day (2014)^61^	13	2	0
Nyström *et al* (2014)^60^	25	7	8
Fisher *et al* (2013)^58^	28	10	0
Moule *et al* (2013)^54^	19	1	3

### Studies that reported improvements in COIL capability

The attributes of successful interventions, along with reported facilitating and hindering factors, were grouped in four themes; (1) engaged managers with a strategic approach; (2) external training and guidance to develop internal knowledge, skills and confidence; (3) process and structure to achieve improvements and learning and (4) individuals and teams with autonomy, accountability, and safety.

#### Engaged managers with a strategic approach

How managers acted was described to some extent in most of the studies. In interventions where COIL capability increased, managers acted strategically by choosing objectives that aligned with overarching organisational goals^33^ and were perceived as meaningful for the employees^34 35^. Interventions were prepared and planned, based on an understanding of the situation, and sometimes also planned to start small and then expand^36^. Managers allocated resources, mainly in the form of time for staff to train and participate in the intervention work^37^, and the approach could be explicitly adapted to be time-effective and cost-effective^38^. Key stakeholders were involved, at least to some degree, for example, clinicians^39^ and patients^40^. In some of the studies with a larger scope, managers also considered multiple levels of the organisation or process, with a systems view^41–43^.

An overwhelming amount of the factors that were described as hindering the interventions was related to the lack of management support and strategy^44^, and not having enough resources^36^, as well as the lack of clear goals and expectations^33^, and lack of prioritisation between projects^21^. The involvement of different stakeholders^38^ and a systems view^45^ were among the most often described facilitating factors. Engaged and strategic managers were also commonly mentioned^46^ as well as the planning of the intervention with a shared understanding and shared objectives^47^.

#### External training and guidance to develop internal knowledge, skills and confidence

One common attribute of interventions that increased COIL capability was the use of external support in the form of coaching, facilitation, training or other expert knowledge. Coaching and facilitation in particular were mentioned in several studies, with three of the studies focusing on different aspects of facilitation and coaching.^48–50^ Important attributes of an effective coach/facilitator were to provide guidance through the change process, to address resistance, and being aware of their own process and how it affects the individuals/teams they work with. Tailoring the approach was important for coaching, facilitation and training^49^, and for the methods and tools that were used^51^.

In many of the studies, some of these expert skills were developed internally in the organisation, with employees acting as coaches, trainers or mentors either from the beginning of the intervention^42^ or after some time and training had passed^39^. Internal champions could also emerge from the groups of employees without external guidance, to lead the projects within the groups^52^.

The skills related to coaching and facilitation were among the most often described facilitating factors^53^. The lack of these skills, or not using the available expertise, were also commonly described as hindering factors^44^.

#### Process and structure to achieve improvements and learning

The studies used various methods for improvements and/or learning, such as the PDSA/PDCA cycle^54^ and experiential learning^55^. Some only reported using a single method, for example, the krAft methodology,^34^ while others reported on several methods or the adoption of an entire approach, for example, lean management.^45^ Common aspects of these methods that were mentioned were reflection^21 49 53 56–58^, and conducting small tests of change/experiments^40 47 49 54 59 60^.

Learning about the methods and how to use them were most frequently achieved by actively using the methods, that is, learning by doing^61^. Some of the interventions consisted of developing training programmes.^37 51 55 62 63^

When conducting the work for improvements and learning, they had meetings and forums for communication and for sharing their progress and learnings with each other and others^42^. They followed up and evaluated the results from their work^21^. Some also reported that good initiatives were praised.^33 39 52^

The use of structured, systematic methods with a prototype/experiment approach^56^ was among the most common facilitating factors. Other common facilitating factors were related to learning by doing^62^, the use of reflection^40^ and meetings and forums for communication 37. Communication failures^43^ were mentioned as a hindering factor, as well as issues with measurements and reports^46^ and technical issues.^63^

#### Individuals and teams with autonomy, accountability and safety

Most of the work within the interventions was conducted in multidisciplinary teams^62^. For some of the studies, teamwork was a focus for the intervention.^63–65^ Some teams had designated team leaders^33^. Attributes concerning individuals were a sense of accountability and commitment^61^, along with confidence to conduct the work^56^ and enough autonomy to be able to conduct it^65^. There was a reported need for an environment that is safe and supporting, with a high degree of psychological safety where behavioural change is supported.^53 56 58^

The individuals’ will to improve^53^, sense of accountability^65^ and ability to perform the tasks^56^ were mentioned as facilitating factors, while lack of buy-in^43^, blaming problems on others^65^ and lacking writing skills^60^ were seen as hindering interventions. Multidisciplinary teams^58^ and individuals or groups as the driving force^52^ were also commonly mentioned as facilitating factors.

### Studies that did not report any improvements in COIL capability

The two studies that did not report an increase in COIL capability generally shared the same attributes as the interventions that did, for example, focusing on teamwork^31^ and the use of a method such as the PDSA cycle.^32^ Some exceptions were that the allocated resources were being decreased in the unsuccessful studies, that plans were made but not followed through, that priorities for the unit and organisation were not aligning with each other, that there were differing perceptions of the goal^31^ and that there existed frustration which was noticed but not addressed.^32^ In the study by Waring *et al*,^32^ there was also a focus from management to overcome change fatigue and change the culture of the organisation, with initial promotional activities including a pledge campaign that was not evident in the other reports.

These two studies reported one-fifth of all the hindering factors, with the majority concerning factors related to the managers and the strategy. There was a lack of prioritisation and support from managers, who made short-term decisions and jumped quickly to new projects, prioritised budget over quality and did not provide adequate resources for the interventions or include all relevant stakeholders.^31 32^ Waring *et al* 32 also reported some facilitating factors: frontline clinicians were supportive, individuals acted as a driving force, and they spread results within the organisation.

## Discussion

The aim of this study was to explore common attributes of interventions that contribute to the development of COIL capability in healthcare organisations and to explore possible facilitating and hindering factors. In this systematic review of 36 studies, four themes were found regarding how this can be achieved; (1) engaged managers with a strategic approach; (2) external training and guidance to develop internal knowledge, skills and confidence; (3) process and structure to achieve improvements and learning and (4) individuals and teams with autonomy, accountability and safety. Both attributes of the interventions and factors that were mentioned as facilitating or hindering were found within these themes.

The findings in this review are in line with previous studies by Loper *et al* 26 on approaches for building quality improvement capacity, by Kaplan *et al* 27 about the influence of context on quality improvement success in healthcare and Lyman *et al* 66 who looked at OL in hospitals.

The results from this review indicate that there is a close connection between the attributes of interventions that succeed in increasing capability for COIL and the different factors that might facilitate or hinder these interventions. One of the attributes of successful interventions, as well as a facilitating factor, was the planning of the intervention with the development of shared objectives and goals and a tailored approach^47^. In some studies, there was also an initial self-assessment prior to the intervention^46^. Additionally, a large amount of the factors that were mentioned as hindrance came from the two studies that did not succeed in increasing COIL capability.^31 32^ This could suggest that some of these factors are addressed when designing successful interventions, or it could be that these are the areas that both researchers and designers of interventions have in mind when designing, evaluating and studying this type of intervention.

A recently published scoping review looked at internal organisational attributes that contribute to improvement and learning capability.^67^ The authors found five organisational attributes that each had associated facilitators and barriers: perceived leadership commitment, open culture, room for team development, initiating and monitoring change and strategic client focus.^67^ These themes show many similarities with the results of this review, aside from client focus not being as prominent in this review as in the review by de Kok *et al*.^67^

Organisational or contextual factors seem to affect the development of COIL capability in healthcare organisations in a powerful and complex manner. The impact of their facilitating or hindering effects is likely to vary within an organisation and over time. For those who wish to increase COIL capability, it could, therefore, be wise to consider the state of these factors within their organisation, since it might not be possible to increase COIL capability if there are too much of the hindering factors or too little of the facilitating factors present.

### Strengths and limitations

It is important to acknowledge the limitations of this review. Most of the studies included in the analysis were not specifically focused on COIL capability, which may have limited the depth of understanding in this area. Additionally, the lack of comparative studies limits the ability to establish causal connections between the identified attributes and intervention outcomes. Furthermore, the search was limited to only English-language and Swedish-language publications, potentially excluding relevant information published in other languages. Another potential limitation is publication bias from unsuccessful interventions not being reported, but a strength of this review is that two unsuccessful studies were found and included. The keywords used for searching the literature were chosen to find as many relevant studies as possible, but the searches might have lacked relevant terms that the author was not aware of and did not find in the preliminary searches. Finally, data extraction and synthesis were performed by a single reviewer, but a research team with researchers from different research fields performed the analysis.

### Suggestions for future research

Further research is needed to investigate what approaches to enhancing COIL capability in healthcare organisations are most effective, and how they are affected by, and connected to, contextual factors and organisational attributes as well as individuals’ choices to convert their capabilities into actions. Comparative studies could be conducted to compare the effectiveness of different approaches, and longitudinal studies to explore the long-term effects of interventions on COIL capability. Important aspects to include in the studies would be the influence of leadership and management, cost-effective strategies for developing internal expert skills needed to train and coach more employees, what the key components of methods and tools are, how to develop psychological safety, as well as well-functioning teams with motivated individuals, and the importance of explicit client focus.

## Conclusion

This systematic review adds to the existing knowledge on interventions that increase COIL capability in healthcare organisations by connecting the attributes of successful interventions with factors that facilitate or hinder those interventions. The findings highlight the importance of engaged managers with a strategic approach, external training and guidance, structured processes and the empowerment of teams and individuals as key aspects of successful interventions. These findings emphasise the importance of prioritising and integrating COIL capability development strategies in healthcare organisations seeking to address both current and future challenges. Future research should further explore the identified attributes and factors to gain a more nuanced and comprehensive understanding of their importance, the interplay between them and explore novel approaches to enhance COIL capability. These findings have practical implications for healthcare leaders, policymakers and researchers, guiding them towards evidence-based strategies that can help healthcare organisations increase their capability and capacity to drive sustainable improvements in care quality, patient safety and organisational performance.

## Data Availability

Data are available upon reasonable request.
