# Supplementary material for: Enhancing capability for continuous organisational improvement and learning in healthcare organisations: a systematic review of the literature 2013–2022
Source: BMJ Open Qual. 2024 Apr 2;13(2):e002566. doi: 10.1136/bmjoq-2023-002566 (PMC10989174; doi:10.1136/bmjoq-2023-002566)
Supplement: Supplementary data [file bmjoq-2023-002566supp001.pdf]

Appendix 1. Overview of the included studies

Author (year)	Country	Unit studied	Type of study	Intervention
Glasheen et al. (2022)	USA	The University of Colorado School of Medicine and College of Nursing, Children's Hospital Colorado (CHCO) and the University of Colorado Hospital UCH). 1960 participants.	Mixed methods: pre-/post-training comparison, and publicly reported quality scores. 2012-2020.	Institute for Health care Quality, Safety and Efficiency (IHQSE)
Källman et al. (2022)	Sweden	62 units in one hospital. Units with at least 10 staff.	Retrospective cross-sectional design. National survey on patient safety culture.	Green Cross instead of other incident reporting system
Myren et al. (2022)	The Netherlands	Department of Gynecologic Oncology of an academic hospital. Patients participated in the meetings.	Prospective study. 2017-2020. Participatory action research approach.	Using a cyclic workflow (PDCA) to improve implementation
Peet et al. (2022)	Australia	Registered nurses. A medical- surgical ward in a hospital.	Observations and interviews several months before the intervention. 1 year. Collaborative and inclusive approach, insider nurses and outsider researchers, action-learning sets.	Emancipatory Practice Development (ePD)
Sweeney et al. (2022)	USA	More or less effective external facilitators working with practices. 23 more effective, 13 less effective.	Sequential mixed methods design. Sep 2015 - Dec 2021.	More effective facilitators compared to less effective
Choo et al. (2021)	USA	7 project teams initially but 2 dropped out. 33 participants in five design for Six Sigma projects conducted in different behavioral healthcare organizations.	Longitudinal survey design spanning 31 weeks.	Reflection on surprises during QI projects (Design for Six Sigma)
Damschroder et al. (2021)	USA	17 obesity treatment teams within the Veterans Health Administration.	Pre- post- evaluation with surveys that assessed team organizational readiness for implementing change and self-rated QI skills. 21 weeks.	LEAP program to increase frontline teams' quality improvement capability
Penney et al. (2021)	USA	12 matched US Veterans Health Administration primary care clinics that did or did not receive coaching. 6 coached, 6 non-coached.	Mixed methods evaluation. 18 months. Clinics were randomized to receive coaching and an online care coordination toolkit or access to the toolkit only.	Distance coaching vs no coaching
Steensgaard et al. (2021)	Denmark	4 nurses and 4 nursing assistants at the Spinal Cord Injury Center. Patients participated.	Action research study. 2 years.	An action research process to engage nurses in development

Walunas et al. (2021)	USA	226 small primary care practices receiving external facilitation.	Retrospective study of different facilitating activities.	Different activities and strategies for practice facilitation
Arora et al. (2020)	USA	15 Federally Qualified Health Centers in a quality improvement collaborative.	Mixed-methods study.<br>18 months.	Improving Clinical Flow Collaborative
Lefebvre et al. (2020)	Canada	The obstetrical unit of the Queensway Carleton Hospital in Ottawa, Ontario.	Pre- /post- evaluation with SCORE survey, program effectiveness tool, and Process and Care measures.<br>12 months.	Education program (including medico-legal risk)
Petit dit Dariel & Cristofalo (2020)	France	70 participating multi-professional staff. 3 units in one hospital, and 2 units in another hospital, having participated in PACTE (*Programme d'amelioration continue du travail en equipe*).	A longitudinal, microlevel ethnographic study. 2015-2019. Field notes, observations, interviews, document analysis.	PACTE (Programme d'amélioration continue du travail en équipe), an experimental program aimed at improving teamwork
Rattray et al. (2020)	USA	External facilitators and multidisciplinary team members in 6 Department of Veterans Affairs medical centers.	An observational, qualitative evaluation of how team members used a web-based Hub. Nested within a prospective, step-wedge implementation trial. A 1-year baseline phase and 1 year of active implementation.	A virtual "Hub" dashboard that provide performance data, a resource library, and a forum for sharing QI plans and tools
Sarff & O'Brien (2020)	USA	A 600-bed academic safety net hospital.	Case study. 3 cohorts between 2016 and 2018. Self-reported QI proficiency assessed through surveys pre, post and 6 months post-Academy.	Quality Academy Program
Dixon & Wellsteed (2019)	United Kingdom (England)	Team A: nurses in an 88-bedded inpatient orthopedic surgery unit, Team B: doctors, nurses, therapists, mental health support workers and administrators in an 18-bedded inpatient ward caring for elderly people with mental health conditions. A senior nurse served as coordinator of the QI work in each team.	Before and after prospective study.<br>1 year.	To emphasize the need for teamwork on a QI project, stages in the QI model were explained using an acronym, A–TEAM.
Gerrish et al. (2018)	United Kingdom	Sheffield Teaching Hospitals NHS Foundation Trust (STH) and local healthcare partners in the Sheffield Falls Care Pathway.<br>The project team comprised	An independent evaluation team comprising two academic/healthcare staff worked with the project team to capture learning. Focus group discussions,	The clinical microsystems (CMS) methodology to develop an

		a project lead (a nurse with extensive experience of older people's services) and three facilitators (a community matron and two physiotherapists with experience of falls services).	individual interviews and observation of a range of meetings in each phase of the initiative. Interview/focus group transcripts and field notes of meetings were analyzed by the evaluation team and the findings discussed with the project team to draw out the learning. 2,5 years.	integrated falls pathway
Nyström et al. (2018)	Sweden	A regional research and development (R&D) unit, and two municipalities handling care of the elderly and people with functional impairments. Convenience sampling of organizational cases was applied to strictly include cases motivated to develop their capability for continuous organizational improvement and learning.	Case study. A multilevel strategy, development loops of five flexible phases, and an action-learning loop. Multiple qualitative methods, i.e., repeated interviews, process diaries, and documents, provided data for conventional content analyses. 28 months.	Sustainable Improvement and Development through Strategic and Systematic Approaches (SIDSSA)
Hefner et al. (2017)	USA	The Ohio State University Wexner Medical Center (OSUWMC), comprising 6 hospitals and 2 campuses.	Survey to measure dimensions of safety culture prior to CRM training and again within 2 years of the initial survey.	Crew resource management (CRM)
Morgan et al. (2017)	United Kingdom	A 75-bed neuroscience ward of a tertiary referral center within a university teaching hospital/trauma center.	Process (compliance with hourly visiting to patients by staff) and outcome (incidence of falls) measures were recorded pre- and postintervention. 12 months.	Staff-led quality improvement intervention
Pannick et al. (2017)	United Kingdom	7 multidisciplinary teams on medical wards in 2 hospitals.	18 months of ethnography and two focus groups were conducted with staff taking part in a trial of prospective clinical team surveillance (PCTS).	Prospective clinical team surveillance (PCTS)
Rydenfält et al. (2017)	Sweden	An action-oriented multidisciplinary group at a hospital operating unit, working according to a methodology built on krAft. The operating unit, the group within the unit, and the project initiated by the group.	Action research. Case study. Mixed methods design. Participatory observations, and individual interviews. As the process leader and the observer in various ways worked with and interacted with the practitioners directly, they also were the primary ''action researchers'' of the project. 14 months.	The krAft methodology
Stelson et al. (2017)	USA	140 staff who had undergone lean training in a	Quantitative regression analysis was performed on Likert-scaled survey	Employee-driven healthcare CI projects

		not-for-profit, community-run, 48-bed hospital.	responses.<br>Qualitative thematic analysis was performed on open-ended survey responses and written reports on CI projects.	
Waring & Crompton (2017)	United Kingdom (England)	An English National Health Service (NHS) hospital.	Case study. The study developed an ethnographic account of the adoption and use of social movement ideas within the hospital. Data collection was carried out over 12 months involving non-participant observations, semi-structured interviews, focus groups and documentary analysis.	Purposeful adoption of social movement ideas in the implementation of a QI strategy
McNamara et al. (2016)	Ireland	Beaumont Hospital.	Interviews about active QI work streams, feedback from learning sessions. 9 months.	On-site interdisciplinary QI learning collaborative
Restrepo et al. (2016)	Canada	25 people and one manager at the internal medicine unit at Hôtel-Dieu de Montréal, a Centre Hospitalier de l'Université De Montréal (CHUM) hospital.	Action research perspective. 15 weeks.	The five-star process (STARS)
Tibor et al. (2016)	USA	The Department of Radiology at Mayo Clinic in Rochester comprised of nearly 1000 employees.	Description of the model and results. A post-project survey of 50 project team members. 6 months.	A collaborative learning approach for process improvement
Fieldston et al. (2015)	USA	2 inpatient units with normal patient loads and workforce within a children's hospital.	Evaluation of a pilot model. 2 months + information from 3 years after the pilot.	Innovation Units for embedding rapid-cycle improvement capabilities
Hellström et al. (2015)	Sweden	Skaraborg Hospital (SkaS).	Action research approach. 2 external academic researchers, 2 insider action researchers. Transformation that occurred from 2003 to 2011. Gathered data over a 5-year period (2005-2010).	Adopting a management innovation
McGrath & Blyke (2015)	USA	Dartmouth-Hitchcock: level 1 trauma center, children's hospital, >1 500 primary and specialty care providers. >10 000 employees were trained.	Evaluation of the development program. 20 months.	Performance improvement competency development program: the Value Institute (VI)

Rangachari et al. (2015)	USA	2 intensive care units within an academic health center. Both units had low baseline adherence to central line bundle and higher-than-expected catheter-related bloodstream infections.	A prospective study. 52 weeks.	Periodic top-down QI communication
Simons et al. (2015)	The Netherlands	A radiotherapy institute.	Triangulation: two surveys were distributed three times, workshops were performed twice, data from an incident reporting system (IRS) was monitored and results were explored using structured interviews with professionals. Averages, chi-square, logistical and multi-level regression were used for analysis. 3 years (2011-2013).	Lean management
Day (2014)	United Kingdom	2 wards with higher numbers of preventable falls, acquired pressure ulcers and underachieving in patient experience surveys, within a hospital.	Before and after-study. 3 months.	ENGAGE and focus group work
Nyström et al. (2014)	Sweden	A hospital. Respondents were unit managers, change facilitators and improvement team members.	Several methods and data sources, including surveys, observations, interviews, process diaries and documents.<br>The aim was to enable theoretical rather than empirical generalizations. 2007-2009.	Two QI programs being implemented at the same time
Fisher et al. (2013)	USA	Family physician professional organization, local healthcare organizations, and individual physicians and practices, in the learning initiative. Volunteers.	A year-long implementation and evaluation project.	Engagement of Groups in Family Medicine Board Maintenance of Certification
Moule et al. (2013)	United Kingdom	4 innovation projects within the Pacesetters Programme. Projects that had completed the PDSA cycle.	Participatory evaluation approach.<br>4 case studies.	The Plan-Do-Study-Act (PDSA) model
